# Mr. Fogo's Observations on Twin Cases

**Published:** 1812-05

**Authors:** A. Fogo

**Affiliations:** Newcastle


*To the Editors of the Medical and Physical Journal.*

GENTLEMEN,

IN No. 156, a correspondent (T. E. B.) says, "in No. 150 is a letter by Mr. Fogo, containing some remarks for which I am desirous of farther authority than his mere *ipse dixit.*" I cannot understand what he means by "farther authority." In making the remarks he alludes to, I wrote my sentiments, which I am inclined and ready to support. Does T. E. B. suspect my veracity? where is he to find farther authority? If he chuses to apply to my friends, or to my brethren and fellow labourers, he may discover whether the practice I have recommended has been successful or the reverse. I will trust both parties; they will "nothing extenuate, or set aught down in malice."

T. E. B. says, "F. is in the habit of delivering the second *per artem.*" He is so, which he (T. E. B.) says is a practice not usually recommended. I know that too. If I have been successful, why should I take any other practitioner's *ipse dixit?*

*dixit?* I have recommended the practice from its safety, ease, and the comfort, it affords to all the parties concerned. If Burns advised to wait three or four hours, and many eminent practitioners in London inculcate the same doctrine; if the gentleman of extensive practice, he mentions, waits *twelve hours* without any ill consequence ensuing; they are only successful. Success is only success, take which method you please. The two methods, the one by an easy and safe application of the obstetric art, the other leaving every thing to nature, may be both successful, but what is gained by waiting *twelve hours?* Nothing good that I can perceive; but the woman and all her friends suffer twelve hours of serious anxiety, which might have been obviated by an early application of the art, in twelve minutes. Further, if no efficient pains of labor appear at the end of twelve hours, what is the practice in London? Have the practitioners recourse to deliver *per artem?* If so, I say they are guilty of great cruelty to the woman and her friends, for their twelve hours' anxiety, at a time which at best is attended with anxiety enough.

Did T. E. B. ever witness the strong and rapid contraction of the os uteri and other parts in the space of twelve hours? If the labor pains return, the woman must suffer almost as much pain as in the first birth. If they do not return, the operator must proceed to apply his art, which is another piece of cruelty which might have been prevented. The second, to my knowledge, may in some cases be extracted unknown to the woman or her attendants, when done immediately.

I told a short story in the same paper, where the delay of delivery was protracted to about sixty hours after the birth of the first child, which terminated in the death of two full-grown boys, who by delay and mismanagement were condemned to die *in utero*, and which, even at the end of forty-eight hours might have been saved, but with greater ease and safety soon after the first.

T. E. B. next says, "opposing the passage of the child is a measure I would strongly recommend, having known syncope and irregular contraction of the womb take place from its too sudden expulsion." I never witnessed such circumstances from such a cause; nor can he have any *authority* to prove the cause and effect. I have witnessed syncope succeed laborious labors; and opposing the passage of the child is a very convenient and effectual method of making an artificial protracted laborious labor; and, whoever the man was who invented and recommended such absurd practice, must have had very little feeling for his fellow creatures; but but it is a companion to the other insignificant practice of guarding the perinæum. If guarding the perinæum, and pushing the child inwards when the woman's natural efforts would have forced it outwards, are the *summum bonum* of London practitioners of midwifery, I must beg leave to differ with them: I always considered the shortening the labor as the duty of every man, instead of protracting it. If he protracts it, he cannot be said (according to the definition) to assist a woman in childbirth; and he appears to me, sitting guarding the perinæum, calling to the woman when the pain returns, to suppress it by "talking, singing, or counting twenty,*" to be of no use, and might safely resign his chair to the lady's maid, if he would merely show her where to place her hand.

In T. E. B.'s quotation from my paper, No. 150, "I may further presume to add, if he (the Essex gentleman) had been of his present opinion, and left the second to nature other two hours, he would very probably have witnessed the death of both mother and child." T. E. B.'s reasoning on the subject is not remarkably scientific. He says, "at the time of his delivering the second child, the effusion of blood *already taken place* must have *nearly* filled the uterus, even supposing it to have not been partially contracted, and by pressing on the mouths of the vessels must have prevented any further hæmorrhage." There was no further hæmorrhage after the seven or eight pounds of blood was allowed to escape: the woman was nearly exanimous. Is this the improved way of suppressing hæmorrhage in London? to allow the uterus to be filled with blood for the sake of pressing on the mouths of the vessels; and to prevent its escape by plugging up the vagina? Do they (the practitioners) consider how much blood a recently-emptied torpid uterus will contain before the blood can sufficiently press on the mouths of the vessels? This is an attempt to suppress hæmorrhage by hæmorrhage, or, according to the London practice, leaving every thing to nature. I am inclined to maintain my first opinion, that, if the second had been delivered immediately after the first, the "deluge of blood" would have been very much lessened or prevented. I have always considered plugging the vagina as a very unscientific method of suppressing or preventing uterine hæmorrhage.

The practice of leaving every thing to nature is something new. I have been told it originated in Dr. Hunter, and who, I have heard, had occasion to change his practice. Does the physician leave fever to nature? Does the surgeon leave fracture,

---

* London Practice of Midwifery.

fracture, dislocation, &c. to nature? There seems nothing in the various branches left to nature but that of midwifery, which in many cases requires as much attention as some of the other branches. The practitioner's attention is solely paid to guarding the perinæum: may not that very elastic part of the system be left to nature, with the other processes of the operation? And let the care of women in labor be undertaken by any discreet, humane, old woman, who never was instructed, as was the fashion formerly. Any woman may guard the perinæum, and there is nothing more required of her. It appears a great imposition that such great fees should be given to a description of men who do no more than any old woman is capable of doing.

I am, &c.

*Newcastle, March* 9, 1812. A. FOGO.

